# Books Reviewed

**Published:** 1866-08

**Authors:** 


### Books Reviewed.

Clinical Notes on Uterine Surgery with special reference to the management of the Sterile condition. By J. Marion Sims, A. B., M. D., etc., etc. New York: Wm. Wood & Co., 61 Walker street, 1866.

Dr. Sims says, "I voluntarily left my own country on account of its political troubles. Our unfortunate civil war continued much longer than any of us, North or South, anticipated. In consequence of this my residence abroad was prolonged far beyond my original intention." It is not very apparent why our political troubles should greatly influence a physician to leave his own country, or remain longer abroad than otherwise agreeable, but during this absence our author has "had time to look over his note books, and to cull such facts as illustrate the method of treating Uterine Diseases at the Woman's Hospital." He says, very modestly, "I wish most sincerely that I could have produced something more worthy of the position so long held by me in that noble charity; for to this I owe all that I know practically of the subjects herein treated."

The followtng conditions the author regards as essential to conception, and treats the subjects under the following general heads:

1—It occurs only during menstrual life.

2—Menstruation should be such as to show a healthy state of the uterine cavity.

3—The os and cervix uteri should be sufficiently open to permit the free exit of the menstrual flow, and also to admit the ingress of the spermatozoa.

4—The cervix should be of proper size, form, shape and density.

5—The uterus should be in a normal position; *i. e.*, neither ante-verted, nor vetro-verted to any great degree.

6—The vagina should be capable of receiving and retaining the spermatic fluid.

7—Semen, with living spermatozoa, should be deposited in the vagina at the proper time

8—The secretions of the cervix and vagina, should not poison or kill the spermatozoa.

Under the first head our author simply tells us that the text is so self-evident that it might be passed without further notice, and then relates several cases of mistakes as to pregnancy, by both physician and patient—of pregnancy in early and advanced life, and closes with the assurance that some of the best men in the profession make most ludicrous mistakes, and that sensible, educated ladies sometimes believe themselves pregnant and are mistaken, are especially apt to be deceived in this way about the turning period of life.

Under the second head he shows that menstruation should be such as to indicate a healthy condition of the uterine cavity, and speaks of scanty menstruation, profuse menstruation, menorrhagia from granular erosion, from fibrous engorgement of the cervix, fungoid granulations, fibrous tumors and uterine polypi. He gives the proper mode of treatment, and speaks of sponge and sea-tangle tents, glycerine, uterine probes, etc., etc. Upon the use of glycerine in uterine surgery he says: "The effect of glycerine thus used is very remarkable. It has great affinity for water. A bit of cotton saturated with glycerine, and exposed to the air, will retain moisture for weeks. When applied to the neck of the womb, as above directed, it seems to set up a capillary drainage by osmosis, producing a copious watery discharge, depleting the tissues with which it lies in contact, and giving them a dry, clean and healthy appearance. When such a dressing is applied to a pyogenic surface on the cervix uteri, for a few hours and then removed, the cut or sore will be as clear of pus as if it were just washed and wiped dry."

Section third is a consideration of the normal and abnormal conditions and relations of the uterus and os tincæ. In section fourth is described the various abnormal conditions of the cervix uteri, with some illustrations, and the method of amputating. Section sixth, is devoted to the various versions and their influence upon steriliiy. Section seventh, to the vaginal imperfections, the nature and properties of semen, spermatozoa, artificial fertilization, period for conception, etc. The last chapter is devoted to the vaginal secretions, vaginitis, leucorrhœa, vaginal injections, etc., etc.

There are a great many points upon which we should be glad to quote our author did space permit. He has given a very full and illustrated account of tumors of the uterus of all forms, and the best means of removing them; also we notice a valuable chapter upon the means of dilating the os uteri. He has made some very sensible remarks upon the use of the uterotome, and though he sometimes uses cutting instruments, he still appears to be quite familiar with the fact that incisions made with the view to enlarge the os uteri, are often quite unsatisfactory—heal over quickly and often only increase, rather than relieve the condition of contraction. We have a word to say upon this operation of enlarging the os uteri by incision, but must defer it and a further consideration of Dr. Sim's book until some future time, merely remarking that every one engaged in the practice of uterine surgery, will be deeply interested in its perusal.

---

Recent advances in Ophthalmic Science; the Boylston Prize Essay for 1865. By Henry W. Williams, M. D., Ophthalmic Surgeon to the City Hospital, Boston; University Lecturer on Ophthalmic Surgery in Harvard University; member of the American Ophthalmic Society, etc., etc. Boston: Ticknor & Fields, 1866.

This essay embraces a great variety of topics, and exceeds the limits of an essay in so much as to almost constitute a complete treatise upon the Diseases of the Eye, the manner of detecting them, and the best mode of cure. The ophthalmoscope and its revelations, and revolutions, constitutes an important part of the work. A careful perusal will show how nearly all that we now regard as knowledge in ophthalmic science is embodied in the recent advances, made in this department, and indicate the immense advance which the introduction of the ophthalmoscope has made in ophthalmology. This essay by Dr. Williams is a compendium of what is known of many of the diseases and accidents of the eye, and their treatment, and too much cannot be said in its favor. It describes the manner of using the ophthalmoscope, tells what may be known by its aid, and how discovered changes may best be rectified or palliated. Physicians having any interest in the diseases and deformities of the visual organs cannot fail to be deeply interested in the perusal of this little volume.

Of the Mechanical Treatment of Chronic Inflammation of the Joints of the Lower Extremities, with the description of some new Apparatuses for producing Extension and Counter-extension at the Knee and Ankle Joints. By L. A. Sayre, M. D.. Surgeon Bellevue Hospital, Professor of Orthopædic Surgery, Bellevue Hospital Medical College, etc., etc. Extracted from the Transactions of the American Medical Association. Philadelphia: For sale at Wm. Wood & Son, and Bailliere Bros., New York. Price $1.00; 1865.

The object of this monograph is to bring before the profession the utility of mechanical appliances in the treatment of chronic inflammation of joints of the lower extremities, with a description of some apparatuses for extension and counter-extension, invented by the writer. The instruments are ingeniously contrived and are eminently adapted for the treatment of the diseases for which they are recommended. It has repeatedly been demonstrated that the beneficial results of mechanical appliances were greatly deprived of their advantage arising from the necessary confinement of the patient, but with the introduction of splints allowing them out-door exercise, orthopædic surgery has achieved a great success. Dr. Sayre, as an orthopædic surgeon, has been prominently instrumental in bringing before the notice of the profession this new method of treatment of chronic diseases of the joints, and we would especially invite their attention to his improved knee and ankle supporters.

---

Asiatic Cholera. By F. A. Burrall, M. D. New York: William Wood & Co., 61 Walker street, 1866.

The book of Dr. Burrall contains a great deal of valuable information, and will prove very interesting and instructive. The author considers the methods of propogation as four in number.

1st—Moist excreta on clothes and bedding of infected persons may be carried by the vapor of water and enter the nostrils and mouth and be swallowed.

2d—Dry excreta on infected clothing may be wafted a short distance by the air when the clothing is moved or unfolded.

3d—Nurses and those who attend the sick may introduce the poison into their system by not washing their hands before taking food.

4th—Utensils used by the sick and not properly cleaned may also contain the germ of the disease.

---

Asiatic Cholera; its Origin and Spread in Asia, Africa and Europe, introduction into America through Canada; Remote and Proximate Causes, Symptoms and Pathology, and the various Modes of Treatment analyzed. By R. Nelson, M. D., Health Commissioner during the first two invasions, 1832, 1834; President of the Medical Board for the District of Montreal. New York: Wm. A. Townsend, publisher, 434 Broome street, 1866.

Seldom has the progress of a malady been watched so eagerly and elicited so much discussion as the scourge which has already appeared in some of our larger cities. Numerous as are the contributions describing the progress, treatment and contagiousness of Asiatic cholera, the profession remains as divided in their opinion as ever.

Dr. Nelson's monograph furnishes us with many interesting facts relating to the spread of cholera in the east, and its propagation on this continent. His connection with the Medical Board of Health in 1832 and 1834, placed him in a most favorable position for observation, and being a carefnl observer his conclusions will n erit the attention of the profession.

---

Biographical Sketches of Distinguished Living New York Surgeons. By Samuel W. Francis, A. M., M. D., Fellow of the New York Academy of Medicine. Reprinted from the Philadelphia Medical and Surgical Reporter. New York: Published by John Bradburn, 1866.

Although we are not disposed to wholly approve of the parading of the accomplishments and achievements of active living members of the profession, still the perusal of these sketches will prove interesting to many of the profession. The memoirs are those of worthy and eminent men, and written in an easy and elegant style.

